# Is Perioperative Hypothermia a Risk Factor for Post-Cesarean Infection?

**DOI:** 10.1080/10647440300025502

**Published:** 2003

**Authors:** Rodney K. Edwards, Kaivou Madani, Patrick Duff

**Affiliations:** Department of Obstetrics and Gynecology University of Florida College of Medicine PO Box 100294 1600 SW Archer Road Gainesville FL 32610-0294 USA

## Abstract

*Objective:* To determine whether hypothermia during Cesarean delivery is a risk factor for postoperative infection.

*Methods:* An historical cohort investigation was conducted on all women delivered by Cesarean at our center during 2001. Initial recovery-room temperature, taken via the oral or axillary route, was used as a surrogate for intraoperative temperature. Adding 0.5^°^C to axillary temperatures generated oral temperature equivalents. Women with chorioamnionitis were excluded, as were those with an initial recovery-room temperature that exceeded 37.9^°^C or was recorded more than 20 minutes after the end of surgery. Prophylactic antibiotics (cefazolin, 1 g) were given during Cesarean delivery.

*Results:* A total of 42 women (7.6%) were diagnosed with postoperative infections. Infections included endometritis (*n*= 25), wound abscess (*n* = 7), wound cellulitis (*n* = 7) and urinary tract infection (UTI) (*n* = 4). No cases of septic pelvic thrombophlebitis or pelvic abscess occurred. One woman had both endometritis and a UTI. Mean temperatures were higher, rather than lower, for women who subsequently had postoperative infections compared with those who did not (36.4 ± 0.8^°^Cvs. 35.9 ± 0.7^°^C;* p* < 0.001). Mean temperatures for the various postoperative infections were as follows: endometritis, 36.5 ± 0.8^°^C (*p*  < 0.001 vs. uninfected group); wound abscess 36.0 ± 0.8^°^C (*p* = 0.63); wound cellulitis, 36.3 ± 0.6^°^C (*p* = 0.14); UTI, 36.7 ± 0.9^°^C (*p* = 0.04).

*Conclusions:*  Women who develop post-Cesarean infections have higher initial recovery-room temperatures than those who do not develop such infections. This suggests the presence of subclinical infection at the time of Cesarean. Evaluating whether intraoperative warming has any role during Cesarean delivery requires a randomized clinical trial.


*Infect Dis Obstet Gynecol 2003;11:75–80*

### Is perioperative hypothermia a risk factor for post-Cesarean infection?

**Rodney K. Edwards, Kaivou Madani and Patrick Duff**

*Department of Obstetrics and Gynecology, University of Florida College of Medicine, Gainesville, FL*

***Objective:*** To determine whether hypothermia during Cesarean delivery is a risk factor for postoperative infection.

***Methods:*** An historical cohort investigation was conducted on all women delivered by Cesarean at our center during 2001. Initial recovery-room temperature, taken via the oral or axillary route, was used as a surrogate for intraoperative temperature. Adding 0.5°C to axillary temperatures generated oral temperature equivalents. Women with chorioamnionitis were excluded, as were those with an initial recovery-room temperature that exceeded 37.9°C or was recorded more than 20 minutes after the end of surgery. Prophylactic antibiotics (cefazolin, 1 g) were given during Cesarean delivery.

***Results:*** A total of 42 women (7.6%) were diagnosed with postoperative infections. Infections included endometritis ($n = 25$), wound abscess ($n = 7$), wound cellulitis ($n = 7$) and urinary tract infection (UTI) ($n = 4$). No cases of septic pelvic thrombophlebitis or pelvic abscess occurred. One woman had both endometritis and a UTI. Mean temperatures were higher, rather than lower, for women who subsequently had postoperative infections compared with those who did not (36.4 ± 0.8°C vs. 35.9 ± 0.7°C; $p < 0.001$). Mean temperatures for the various postoperative infections were as follows: endometritis, 36.5 ± 0.8°C ($p < 0.001$ vs. uninfected group); wound abscess 36.0 ± 0.8°C ($p = 0.63$); wound cellulitis, 36.3 ± 0.6°C ($p = 0.14$); UTI, 36.7 ± 0.9°C ($p = 0.04$).

***Conclusions:*** Women who develop post-Cesarean infections have higher initial recovery-room temperatures than those who do not develop such infections. This suggests the presence of subclinical infection at the time of Cesarean. Evaluating whether intraoperative warming has any role during Cesarean delivery requires a randomized clinical trial.

Key words: PUERPERAL ENDOMETRITIS; WOUND INFECTION; CESAREAN DELIVERY

Cesarean delivery is the most common indication for laparotomy in the USA[1]. In 2001, 24.4% of all deliveries in this country were by Cesarean[2]. Therefore more than one million Cesareans are now performed in the USA each year. The likelihood of post-Cesarean infection varies widely, depending on sociodemographic factors and whether the Cesarean was performed before or after labor and/or rupture of the membranes. The incidence of endometritis after Cesarean delivery is 5–20%[3], and the rate of wound infection is 2–16%[4]. These infections occur despite the use of prophylactic antibiotics. Therefore, each year tens of thousands of gravidas in this country develop post-Cesarean infections.

Intraoperative hypothermia may increase the incidence of postoperative infections. Thermoregulatory vasoconstriction occurs as a result of

Correspondence to: Rodney K. Edwards, MD, MS, Department of Obstetrics and Gynecology, University of Florida College of Medicine, PO Box 100294, 1600 SW Archer Road, Gainesville, FL 32610-0294, USA. Email: edwardsr@obgyn.ufl.edu

© 2003 The Parthenon Publishing Group 

intraoperative hypothermia[5]. Vasoconstriction decreases the partial pressure of oxygen in tissues and decreases resistance to infection in animals[6,7]. The production of free radicals by leukocytes is oxygen dependent over the range of partial pressures of oxygen that are found in wounds[8]. In addition, hypothermia can adversely affect other functions of leukocytes that are involved in destroying microbes, such as chemotaxis, phagocytosis and antibody production[9]. In a guinea-pig model, mild hypothermia during anesthesia has been shown to decrease resistance to infection with *Escherichia coli* and *Staphylococcus aureus*[10,11]. Finally, a randomized controlled trial in humans has demonstrated a decrease in the rate of wound infection in patients undergoing colorectal surgery from 19% in the standard care group to 6% in the group that received additional warming measures ($p = 0.009$)[12].

If intraoperative hypothermia is associated with increased rates of post-Cesarean infections, intraoperative warming might result in a substantial decrease in morbidity and cost of care, owing to the prevalence of Cesarean delivery and the relatively inexpensive nature of the measures that are utilized to warm a patient who is undergoing laparotomy. The objective of this investigation was to evaluate whether hypothermia during Cesarean delivery is a risk factor for postoperative infection.

### SUBJECTS AND METHODS

An historical cohort investigation was conducted on all women delivered by Cesarean at Shands Hospital at the University of Florida from 1 January to 31 December 2001. Patients who underwent Cesarean delivery were identified from the obstetric database maintained by the Division of Maternal–Fetal Medicine. The medical records of these women were obtained and reviewed. The study was approved by the University of Florida Health Center Institutional Review Board.

Demographic data and details of the labor, delivery and postpartum course were abstracted from these records and entered into a relational database (Access 2000, Microsoft Corporation, Redmond, WA). SAS Version 8.0 (SAS Institute, Cary, NC) was utilized for statistical analysis. The unpaired Student's *t*-test was used for continuous data. Categorical variables were analyzed using Chi-square or Fisher's exact test, as appropriate. All statistical tests were two-tailed, and utilized an alpha value of 0.05.

The initial recovery-room temperature accurately reflects intraoperative temperature status[12]. We used the initial recovery-room temperature, taken via the oral or axillary route, as a surrogate for intraoperative temperature. Adding 0.5°C to axillary temperatures generated oral temperature equivalents[13,14]. The mean oral (or equivalent) temperature was the primary outcome variable.

Exclusion criteria were as follows: clinical diagnosis of chorioamnionitis at the time of Cesarean; intrapartum temperature over 37.9°C but no other diagnostic criteria for chorioamnionitis; initial recovery-room temperature over 37.9°C; initial recovery-room temperature recorded more than 20 minutes after the end of surgery. All women received prophylaxis against post-Cesarean infections with cefazolin, 1 g administered intravenously, after the umbilical cord was clamped. For women undergoing Cesarean delivery after labor or rupture of the membranes, a second dose was given 8 hours later. Women with a history of allergy to β-lactam antibiotics or cephalosporins received a single injection of gentamicin and clindamycin instead of cefazolin.

Subjects were considered to have had endometritis if they developed an oral temperature of 38.0°C or higher more than 4 hours postoperatively, had no signs of infection at sites other than the uterus, and were treated with antibiotics for this indication. Those women who were treated with antibiotics during the postoperative period for a 'wound infection' were considered to have either a wound abscess or wound cellulitis. The former category was utilized for those subjects in whom the wounds were opened and purulent fluid was encountered. The latter category was utilized for those subjects who had documented induration and warmth at the wound site but no purulent discharge from the wound. Subjects who had dysuria and a urine culture showing > 100 000 colonies/ml of a single uropathogen were classified as having a urinary tract infection (UTI).

### RESULTS

During the study period, a total of 657 women were delivered by Cesarean. Women were excluded from the cohort because of missing charts ($n = 18$), chorioamnionitis ($n = 55$), intrapartum temperature over 37.9°C but no other criteria for diagnosing chorioamnionitis ($n = 2$), initial recovery-room temperature over 37.9°C ($n = 8$) or initial recovery-room temperature having been obtained more than 20 minutes after the end of surgery ($n = 25$). The cohort consisted of the remaining 555 women. In total, 42 women (7.6%) had postoperative infections. The distribution of these infections was as follows: endometritis, $n = 25$; wound abscess, $n = 7$; wound cellulitis, $n = 7$; UTI, $n = 4$. No cases of septic pelvic thrombophlebitis or pelvic abscess occurred. One woman had both endometritis and a UTI. The demographic data are shown in Table 1, and the data on labor and intraoperative course are presented in Table 2.

Table 3 shows the mean initial recovery-room temperatures for the infected and uninfected groups. The initial recovery-room temperature was taken via the oral route for all but 17 subjects; the latter subjects had their temperatures taken via the axillary route. One of the subjects whose initial recovery-room temperature was taken via the axillary route later developed endometritis. The other 16 subjects were in the uninfected group. Mean temperatures were higher, rather than lower, for women who subsequently had postoperative infections compared with those who did not. Of the eight women who were excluded because their initial recovery-room temperature was over 37.9°C, five developed puerperal endometritis and one developed wound cellulitis.

### DISCUSSION

Our study design has some limitations. During data collection, all subjects' medical records were reviewed no less than 60 days after discharge from the delivery hospitalization. Therefore subjects who developed postoperative infections after hospital discharge and then presented to the emergency department for evaluation were still detected as having postoperative infections. However, subjects who presented to another facility for treatment of postoperative infections would

**Table 1** Demographic data

09 July 2003 14:29:20

| *Variable* | *Infected* ($n = 42$) | *Uninfected* ($n = 513$) | *p-value* |
|---|---|---|---|
| Age (years) | 24.8 ± 6.5 | 27.2 ± 6.8 | 0.03 |
| Height (inches) | 63.9 ± 3.5 | 64.0 ± 2.8 | 0.83 |
| Weight (pounds) | 200 ± 49 | 195 ± 51 | 0.55 |
| Gestational age (weeks) | 37.0 ± 4.4 | 36.7 ± 3.8 | 0.64 |
| Nulliparous | 66.7 | 36.3 | < 0.001 |
| Race | | | 0.68 |
| White | 59.5 | 57.7 | |
| Non-white | 40.5 | 42.3 | |
| Diabetic | | | 0.75 |
| No | 85.7 | 84.0 | |
| Gestational | 14.2 | 13.3 | |
| Pregestational | 0 | 2.7 | |
| Hypertension | | | 0.89 |
| No | 78.6 | 78.4 | |
| Chronic | 2.4 | 2.3 | |
| Pre-eclampsia/eclampsia | 19.0 | 19.4 | |
| Preoperative hematocrit (%) | 33.8 ± 4.1 | 34.1 ± 3.7 | 0.65 |
| Neonatal birth weight (g) | 3039 ± 898 | 3024 ± 925 | 0.92 |
| Male neonate | 48.7 | 55.0 | 0.45 |

Data are presented as mean values ± standard deviation or proportion of *n*. Due to rounding, some category proportions do not total exactly 100%

**Table 2** Data on labor and intraoperative course

| *Variable* | *Infected (n = 42)* | *Uninfected (n = 513)* | *p-value* |
|---|---|---|---|
| In labor | 85.7 | 50.3 | < 0.001 |
| Total number of vaginal examinations | 5.5 ± 4.2 | 2.2 ± 3.0 | < 0.001 |
| Vaginal examinations after ROM | 4.0 ± 3.6 | 1.2 ± 2.1 | < 0.001 |
| Duration of ROM (hours) | 35.1 ± 97.7 | 9.9 ± 66.8 | 0.02 |
| Duration of IM (hours) | 4.6 ± 5.4 | 1.5 ± 3.5 | < 0.001 |
| Meconium-stained fluid | 31.0 | 11.5 | < 0.001 |
| GBS prophylaxis | 21.4 | 17.3 | 0.44 |
| Duration of Cesarean (minutes) | 46.0 ± 14.9 | 48.5 ± 13.8 | 0.27 |
| Emergent Cesarean | 9.5 | 9.2 | 0.94 |
| Intraoperative $FIO_2$ (%) | 31.4 ± 11.5 | 33.5 ± 17.9 | 0.45 |
| Estimated blood loss (ml) | 812 ± 220 | 788 ± 223 | 0.50 |
| Time between end of surgery and initial recovery-room temperature (minutes) | 9.6 ± 5.1 | 8.1 ± 4.6 | 0.07 |
| Skin incision | | | |
| Transverse | 92.9 | 86.3 | 0.23 |
| Vertical | 7.1 | 13.7 | |
| Uterine incision | | | 0.70 |
| Low transverse | 97.6 | 96.5 | |
| Classical | 2.4 | 3.5 | |

Data are presented as mean values ± standard deviation or proportion of *n*. ROM, rupture of membranes; IM, internal monitors; GBS, group B streptococcus; $FIO_2$, fraction of inspired oxygen. The duration of the Cesarean was the difference between the 'begin surgery' and 'end surgery' times as recorded on the anesthesia flow record

**Table 3** Initial recovery room temperatures

Z:\Customer\PARTHEN\IDOG\A4577 - IDOG Vol 11 No 2 - June 2003.vp
09 July 2003 14:29:22

| *Group* | *n* | *Initial recovery-room temperature (°C)* | *Range (°C)* | *p-value* |
|---|---|---|---|---|
| Uninfected | 513 | 35.9 ± 0.7 | 32.5–37.9 | Reference |
| Total infected | 42 | 36.4 ± 0.8 | 34.9–37.9 | < 0.001 |
| Endometritis | 25 | 36.5 ± 0.8 | 35.0–37.9 | < 0.001 |
| Wound abscess | 7 | 36.0 ± 0.8 | 34.9–37.0 | 0.63 |
| Wound cellulitis | 7 | 36.3 ± 0.6 | 35.3–37.0 | 0.14 |
| Urinary tract infection | 4 | 36.7 ± 0.9 | 35.4–37.4 | 0.04 |

Data are presented as mean values ± standard deviation not have been detected. In addition, we cannot account for the effect of type of anesthesia on the initial recovery-room temperature. However, almost all of the subjects in this study ($n = 524$; 94.4%) had their Cesarean deliveries performed under epidural or spinal anesthesia.

Our study failed to show an association between intraoperative hypothermia and an increased likelihood of post-Cesarean infections. In contrast, the uninfected group on average had lower initial recovery-room temperatures. One other study has evaluated the association between intraoperative hypothermia and post-Cesarean infection, specifically wound infection. Munn *et al.*[13] performed a case–control study that compared mean initial recovery-room core temperatures (derived by adding 0.5°C to the oral temperature) in women who had post-Cesarean wound infections and in controls matched for age, weight, presence of gestational hypertension and duration of surgery. In their study, the mean initial recovery-room core temperature was 36.3°C in the women who had wound infections and 36.6°C in the controls ($p = 0.8$). Munn *et al.*[13] speculated that their results may have differed from those in the study of patients who underwent colorectal surgery[12], due to differences in operating time between Cesarean delivery and colorectal surgery (resulting in lower mean intraoperative temperatures in the control group of colorectal surgery patients) and differences between gravidas and patients undergoing colorectal surgery.

Although those factors may have contributed to the differences in the results, the findings of our study suggest a different reason. We found a higher rather than lower mean initial recovery-room temperature in the group that developed post-Cesarean infections compared with the group that did not. This result leads us to conclude that the women who developed postoperative infections had subclinical chorioamnionitis at the time of Cesarean delivery, resulting in a relatively higher mean initial recovery-room temperature. Previous studies have shown that women who develop postpartum endometritis often have positive amniotic fluid cultures at the time of Cesarean delivery[15–17]. In addition, many of the risk factors for chorioamnionitis (number of vaginal examinations during labor, and duration of labor, ruptured membranes and internal monitoring) have also been shown to be risk factors for post-Cesarean wound infection[18].

Because there is no way to adjust for the effect of differences in bacterial contamination rates between patients, evaluation of any possible role of intraoperative warming during Cesarean delivery will require a randomized clinical trial. This type of design would also provide for balance between groups with regard to other potentially confounding factors, such as anesthesia type and volume and the temperature of intravenous fluid administered. Such a trial could evaluate warming via intravenous fluids and/or conduction warmers. If intraoperative warming was found to be effective in decreasing the rate of postoperative infections (as it is in colorectal surgery patients), this approach may be a cost-effective way to improve outcomes for our patients.

### REFERENCES

1. Taffel SM, Placek PJ, Moien M, *et al.* 1989 US Cesarean section rate steadies: VBAC rate rises to nearly one in five. *Birth* 1991;18:73–7
2. Martin JA, Park MM, Sutton PD. Births: preliminary data for 2001. *Natl Vital Stat Rep* 2002;50:1–20
3. Duff P. Maternal and perinatal infection. In Gabbe SG, Niebyl JR, Simpson JL, eds. *Obstetrics: Normal and Problem Pregnancies*, 4th edn. New York: Churchill Livingstone, 2002:1293–345
4. Owen J, Andrews WW. Wound complications after Cesarean sections. *Clin Obstet Gynecol* 1994; 37:842–55
5. Ozaki M, Sessler DI, Suzuki H, *et al.* Nitrous oxide decreases the threshold for vasoconstriction less than sevoflurane or isoflurane. *Anesth Analg* 1995;80:1212–16
6. Chang N, Mathes SJ. Comparison of the effect of bacterial inoculation in musculocutaneous and random-pattern flaps. *Plast Reconstr Surg* 1982; 70:1–10
7. Jonsson K, Hunt TK, Mathes SJ. Oxygen as an isolated variable influences resistance to infection. *Ann Surg* 1988;208:783–7
8. Hohn DC, MacKay RD, Halliday B, *et al.* The effect of $O_2$ tension on microbicidal function of leukocytes in wound and *in vitro*. *Surg Forum* 1976;27:18–20
9. Van Oss CJ, Absolom DR, Moore LL, *et al.* Effect of temperature on the chemotaxis, phagocytic engulfment, digestion and $O_2$ consumption of human polymorphonuclear leukocytes. *J Reticuloendothelial Soc* 1980;27:561–5
10. Sheffield CW, Sessler DI, Hunt TK. Mild hypothermia during isoflurane anesthesia decreases resistance to *E. coli* dermal infection in guinea pigs. *Acta Anaesthesiol Scand* 1994;38:201–5
11. Sheffield CW, Sessler DI, Hunt TK, *et al.* Mild hypothermia during halothane-induced anesthesia decreases resistance to *Staphylococcus aureus* dermal infection in guinea pigs. *Wound Repair Regen* 1994;2:48–56

12. Kurz A, Sessler DI, Lenhardt R. Perioperative normothermia to reduce the incidence of surgical-wound infection and shorten hospitalization. *N Engl J Med* 1996;334:1209–15
13. Munn MB, Rouse DJ, Owen J. Intraoperative hypothermia and post-Cesarean wound infection. *Obstet Gynecol* 1998;91:582–4
14. Marino P. Approaches to nosocomial fever. In Marino P, ed. *The ICU Book*. Philadelphia, PA: Lea and Febiger, 1991:571–87
15. Gilstrap LC, Cunningham FG. The bacterial pathogenesis of infection following Cesarean section. *Obstet Gynecol* 1979;53:545–9
16. Blanco JD, Gibbs RS, Castaneda YS, *et al*. Correlation of quantitative amniotic fluid cultures with endometritis after Cesarean section. *Am J Obstet Gynecol* 1982;143:897–901
17. Yancey MK, Clark P, Duff P. The frequency of glove contamination during Cesarean delivery. *Obstet Gynecol* 1994;83:538–42
18. Gibbs RS, Blanco JD, St. Clair PJ. A case–control study of wound abscess after Cesarean delivery. *Obstet Gynecol* 1983;62:498–501

RECEIVED 10/10/02; ACCEPTED 03/17/03
